# Publisher Correction: Enhanced silica export in a future ocean triggers global diatom decline

**DOI:** 10.1038/s41586-022-05025-0

**Published:** 2022-07-07

**Authors:** Jan Taucher, Lennart T. Bach, A. E. Friederike Prowe, Tim Boxhammer, Karin Kvale, Ulf Riebesell

**Affiliations:** 1 GEOMAR Helmholtz Centre for Ocean Research Kiel, Kiel, Germany; 2 Institute for Marine and Antarctic Studies, University of Tasmania, Hobart, Tasmania Australia; 3 GNS Science, Lower Hutt, New Zealand

**Keywords:** Biooceanography, Element cycles, Climate-change impacts, Marine biology, Marine chemistry

Correction to: *Nature* 10.1038/s41586-022-04687-0 Published online 25 May 2022

In the version of this article initially published, the units listed on the Fig. 3a “Depth (m)” *y*-axis were incorrect (running from “0” to “8,000” and incorrectly ordered) and have been corrected to range from “0” to “6,000.” Additionally, in the section ‘Impacts of OA on Si:N_export_’, the sentence ‘Most probably, lower pH accelerated the dissolution of sinking opal and thereby increased Si:N_export_.’ should read ‘Most probably, lower pH decelerated the dissolution of sinking opal and thereby increased Si:N_export_.’ The changes have been made to the HTML and PDF versions of the article

