# Author Correction: Enhanced silica export in a future ocean triggers global diatom decline

**DOI:** 10.1038/s41586-024-08425-6

**Published:** 2024-11-25

**Authors:** Jan Taucher, Lennart T. Bach, A. E. Friederike Prowe, Tim Boxhammer, Karin Kvale, Ulf Riebesell

**Affiliations:** 1 GEOMAR Helmholtz Centre for Ocean Research Kiel, Kiel, Germany; 2 Institute for Marine and Antarctic Studies, University of Tasmania, Hobart, Tasmania Australia; 3 GNS Science, Lower Hutt, New Zealand

**Keywords:** Biooceanography, Element cycles, Climate-change impacts, Marine biology, Marine chemistry

Correction to: *Nature* 10.1038/s41586-022-04687-0 Published online 25 May 2022

In the version of the article initially published, in the key to Fig. 3b the red and orange lines were switched and have now been amended so that red represents “RCP8.5” and orange “RCP6.0” in the HTML and PDF versions of the article.

